# Special forms in twin pregnancy - ACARDIAC TWIN/ Twin reversed arterial perfusion (TRAP) sequence

**Published:** 2015

**Authors:** FA Anca, A Negru, AE Mihart, C Grigoriu, RE Bohîlțea, A Șerban

**Affiliations:** * Carol Davila” University of Medicine and Pharmacy, Bucharest, Romania; ** Obstetrics and Gynecology Department, University Emergency Hospital Bucharest, Romania; *** Gynecology Department, “Polizu Hospital”, Bucharest, Romania

**Keywords:** pumping twin, acardiac twin, monozygous twin pregnancy, reversed arterial perfusion (TRAP), radiofrequency ablation (RFA)

## Abstract

Twin pregnancy generally represents a high-risk pregnancy, but monozygous twin pregnancy is a real challenge for the obstetrician due to the serious complications that may occur during its evolution. A very rare, severe complication of monozygous twin pregnancy, which we recently dealt with in the Obstetrics and Gynecology Department of the University Emergency Hospital Bucharest, was a monochorionic monoamniotic twin pregnancy with acardiac twin (TRAP). One of the fetuses (acardiac twin) presented a rudimentary unfunctional heart or even no heart at all, underdeveloped inferior part of the body and head, being transfused by the other fetus with a normal heart (pumping twin) by one superficial arterio-arterial anastomosis through which blood pumped backwards. The understanding of these cases is mandatory in order to offer maximum survival and heath chances to the viable fetus.

**Abbreviations**:

RFA = radiofrequency ablation, TRAP = reversed arterial perfusion

## Case report

A Caucasian 35-year-old patient, from the urban area, presented to the Department of Obstetrics-Gynecology for an ultrasound evaluation at 13 weeks and 4 days of pregnancy. A transabdominal ultrasound is performed by using a RAB transducer of 4-8 MHz. A monochorionic diamniotic twin pregnancy of 13 weeks and 4 days is diagnosed together with a TRAP sequence with acardius acephalus twin. 

The normal fetus (pumping twin): normal fetal anatomy for gestational age; among the ultrasound markers for the 1st trimester screening, the ductus venosum had a negative “a” wave (**Fig. 1 a**-**c**). 

**Fig. 1a:**
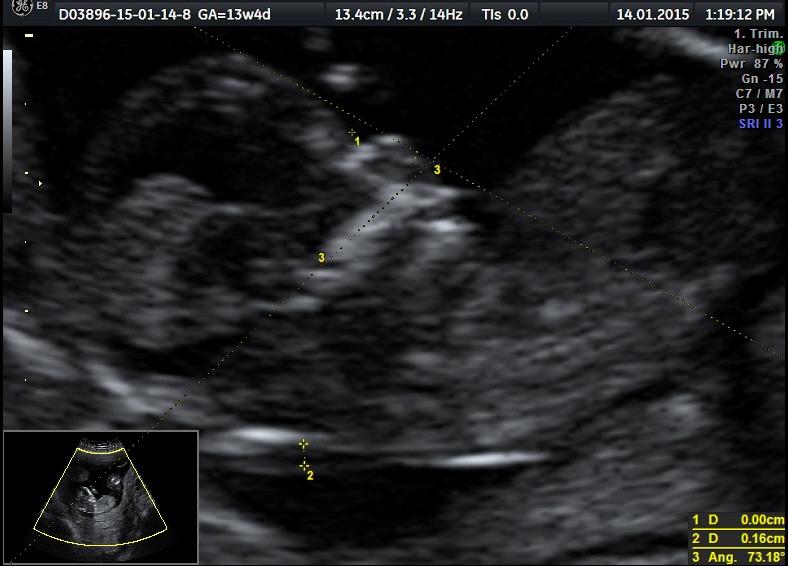
Developed normal fetus (pumping twin): saggital section

**Fig. 1b:**
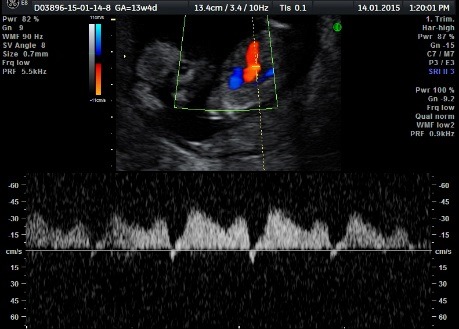
Doppler sonography of the developed normal fetus (pumping twin): ductus venosum having a negative “a” wave

**Fig. 1c:**
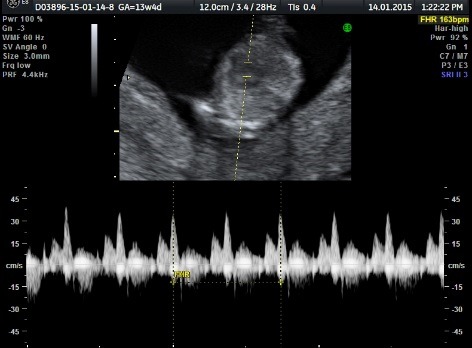
Developed normal fetus (pumping twin): tricuspid flow without regurgitation

Acardiac twin: from the point of view of the fetal anatomy, a heterogeneous tissular mass without abdominal and thoracic organs is distinguished together with an absent cephalic part and diffuse edema. Doppler sonography highlights a present arterial flow (**Fig. 2 a**,**b**).

**Fig. 2a:**
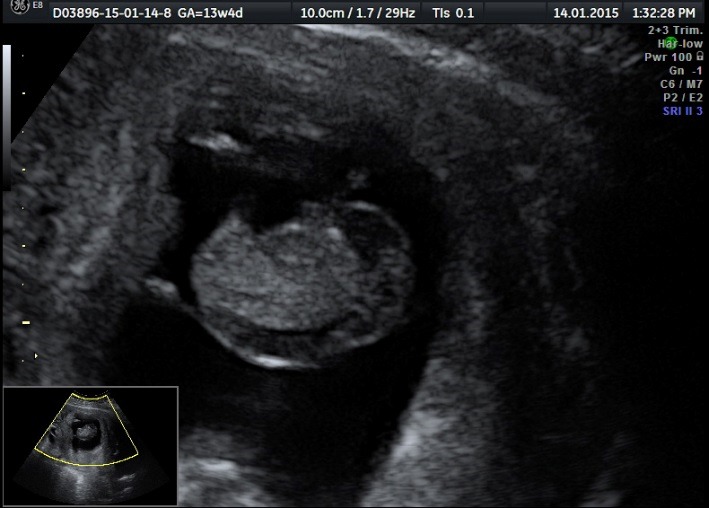
Acardiac twin: heterogeneous tissular mass, no abdominal and thoracic organs can be detected

**Fig. 2b:**
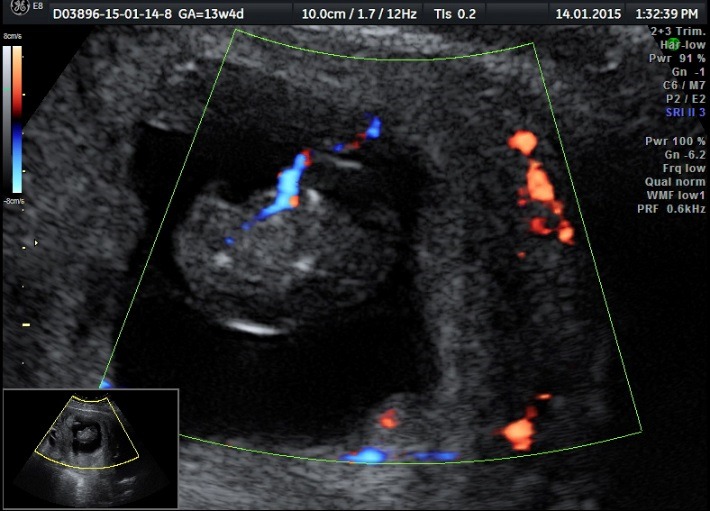
Acardiac twin: absent heart

The patient was counseled according to the international protocols and recommendations specific for that pathology, as far as the risks of the pregnancy were concerned: 

• Risk of chromosomal abnormalities (9%) 

• Risk of involution of the acardiac twin 

• Risk of hemodynamic complications which could lead to the involution of the pregnancy 

• Risk of premature birth, of hemodynamic modifications and of polyhydramnios installation 

• Possibility of intervention and its risks. 

**Fig. 3:**
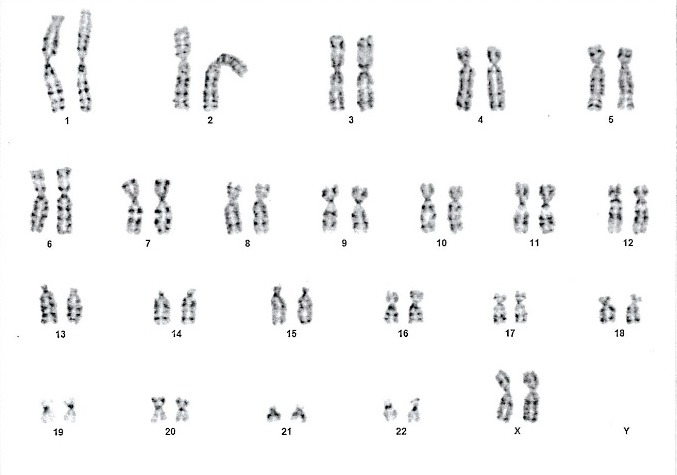
Karyotype

The integrated test for chromosomal numerical anomalies showed a low risk. The cytogenetic analysis of amniotic fluid highlighted a normal feminine karyotype, in accordance with FISH test (**Fig. 3**). 

The patient was periodically monitored by performing serial ultrasound examinations. In addition, at 18 weeks and 4 day of pregnancy, modifications were observed in both fetuses: the normal fetus (pumping twin) presented a normal fetal anatomy and the heart examination was in normal ranges, but what was noticed was the installation of polyhydramnios (**Fig. 4a**-**d**).

**Fig. 4a:**
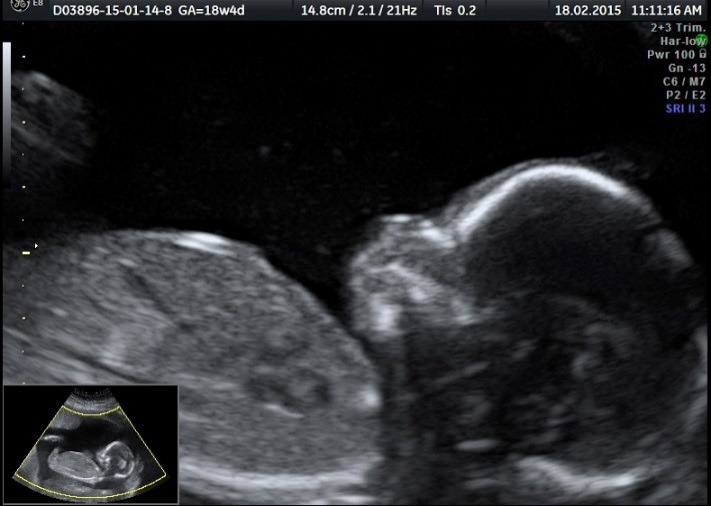
Normal polyhydramnios fetus (pumping twin)

**Fig. 4b:**
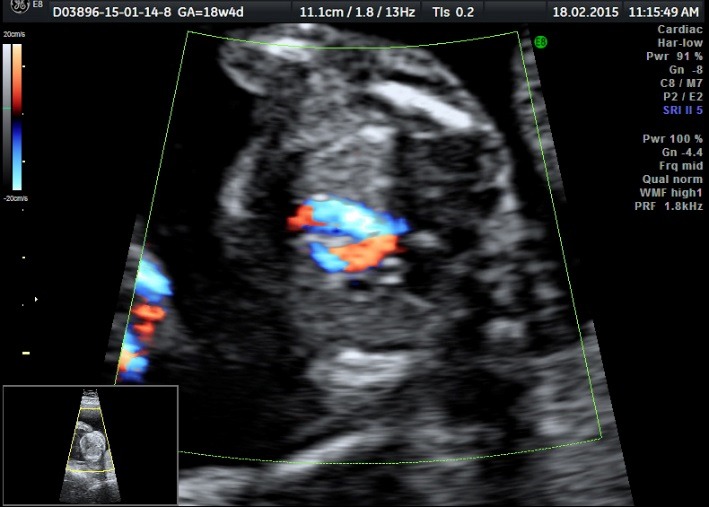
Normal fetus (pumping twin) with a normal heart – Doppler examination of the 3 vassel viewaryotype

**Fig. 4c:**
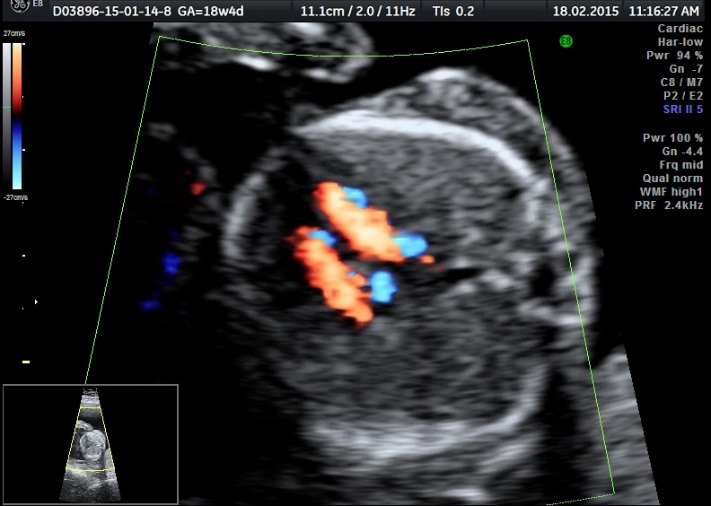
Normal fetus (pumping twin) with a normal heart – Doopler examination of the 4 chamber view

**Fig. 4d:**
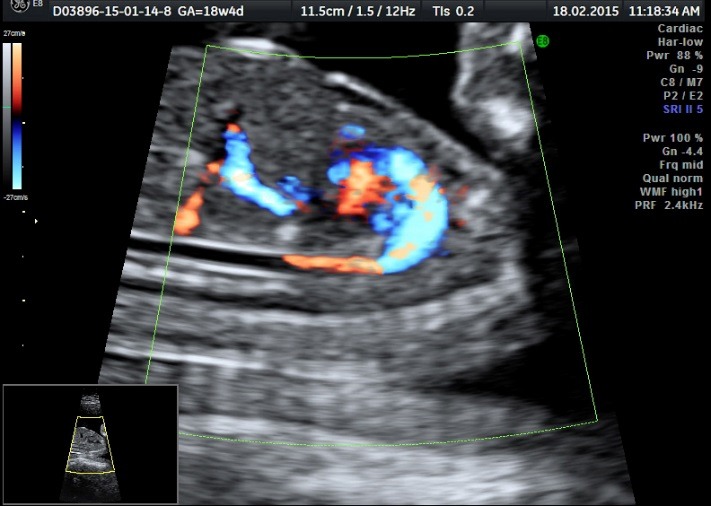
Normal fetus (pumping twin) with a normal fetal blood flow – Doppler examination of the Aortic arch

The ultrasound examination of the acardiac twin highlighted the size of the volume of the tissular mass, an incomplete fetal skeleton, absent abdominal organs, absent thorax, absent cephalic pole, a generalized edema, and normal legs. Doppler sonography presented an umbilical artery with a high resistance index (**Fig. 5 a**-**d**). 

**Fig. 5a:**
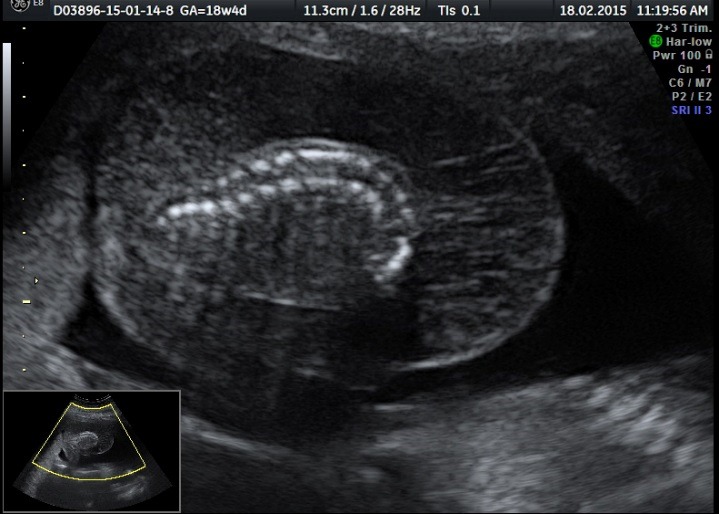
Acardiac twin: incomplete fetal skeleton

**Fig. 5b:**
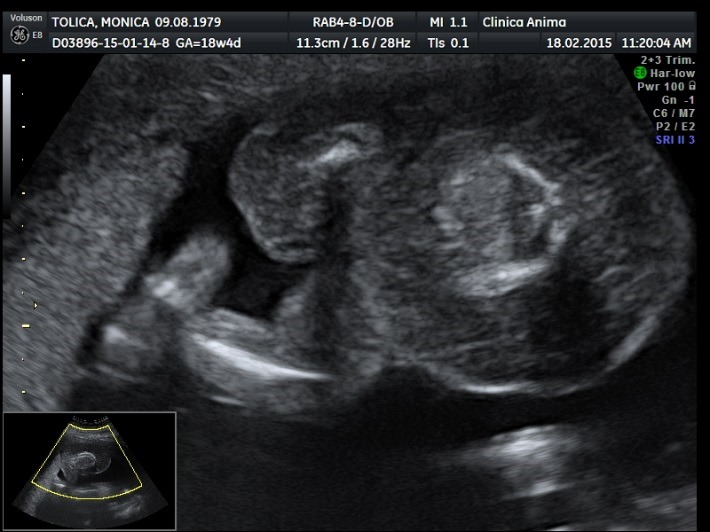
Acardiac twin: normally developed legs

**Fig. 5c:**
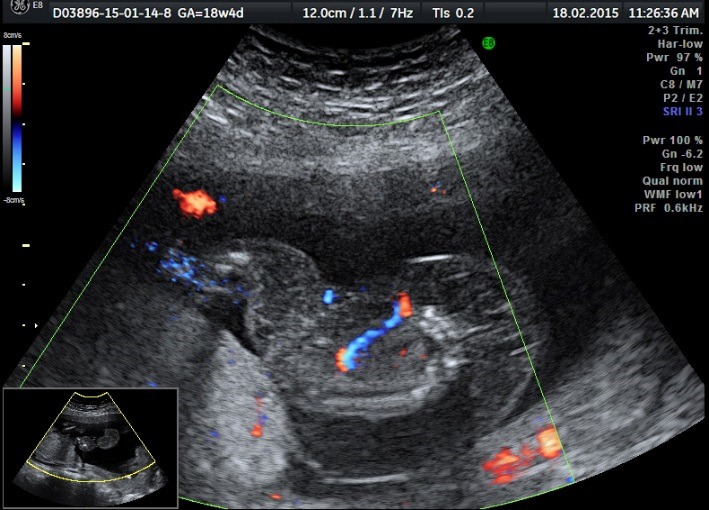
Acardiac twin: absent heart

**Fig. 5d:**
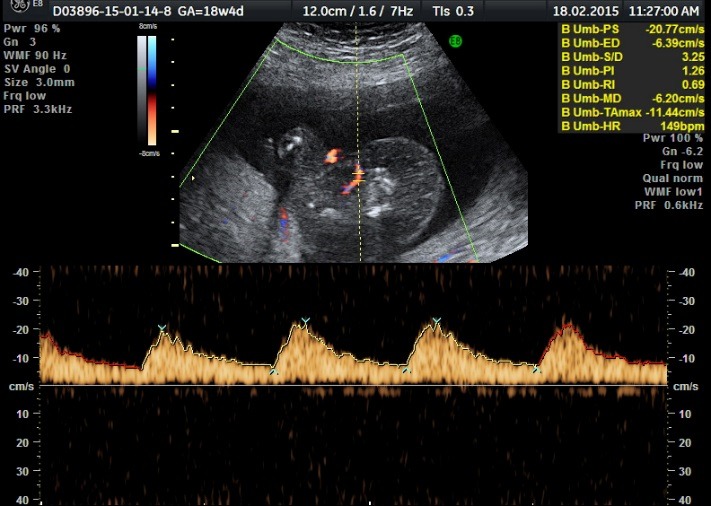
Acardiac twin: Doppler sonography with a high RI index on the umbilical artery

At 18 weeks of pregnancy, we underlined the growth of the acardiac twin mass without cardiac insufficiency signs in the pumping twin, concomitantly with the polyhydramnios installation. 

A fetoscopic surgery was decided to be performed: occlusion of the umbilical cord of the acardiac twin. The evolution of the normal fetus was favorable. 

**Fig. 6:**
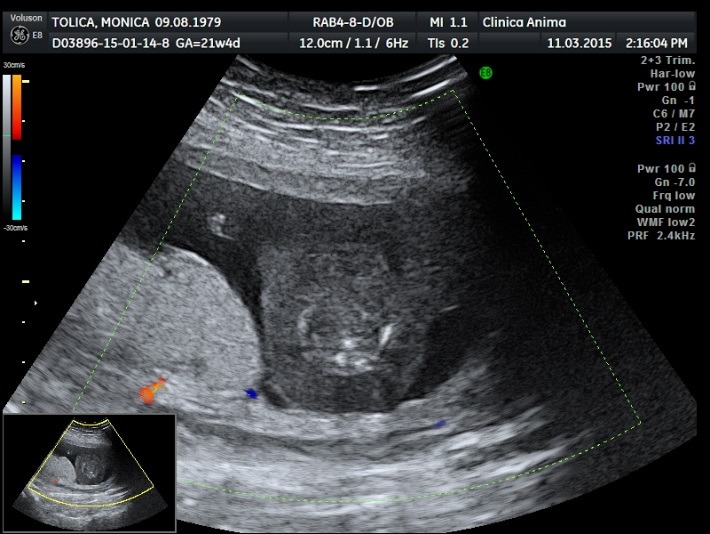
Involution of the tissular mass growth and lack of Doppler signal

The patient came back for a routine ultrasound examination at 22 weeks of pregnancy. The ultrasound examination showed normal structures in the pumping twin, the amniotic fluid being normal as far as the quantity was concerned. Regarding the acardiac twin, an involution of the tissular mass growth and a lack of Doppler signal were noticed (**Fig. 6**). 

The follow-up of the patient was continued by performing ultrasound examinations, all being in normal parameters. 

From the maternal point of view, as a result of the clinical and paraclinical investigations, the appearance of a last trimester eclampsia was outlined. It manifested by hypertension and suggestive modifications of the paraclinical samples, so that, at 36 weeks of pregnancy, it was decided that a low-segment transverse C-section should be performed. An alive female fetus of 3000g was extracted together with the acardiac twin of 300g (**Fig. 7a**,**b**). 

**Fig. 7a:**
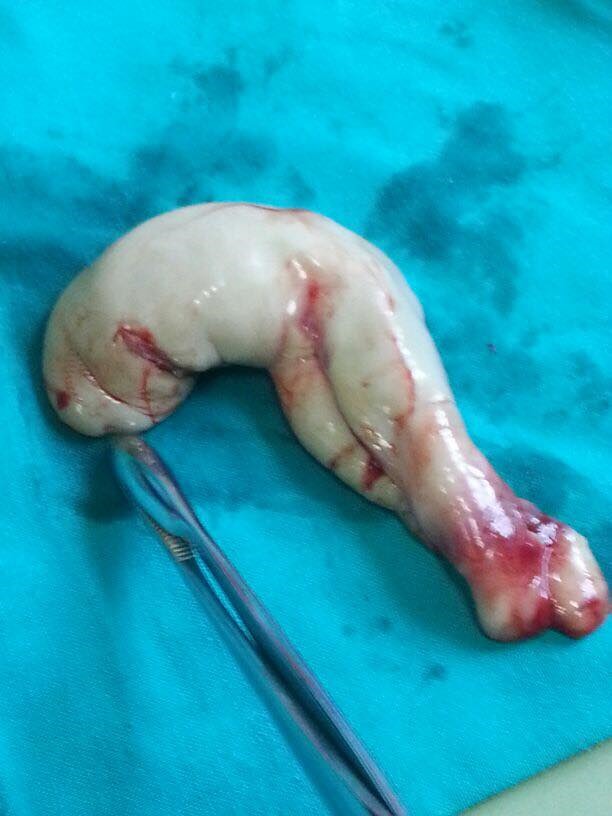
Acardiac twin

**Fig. 7b:**
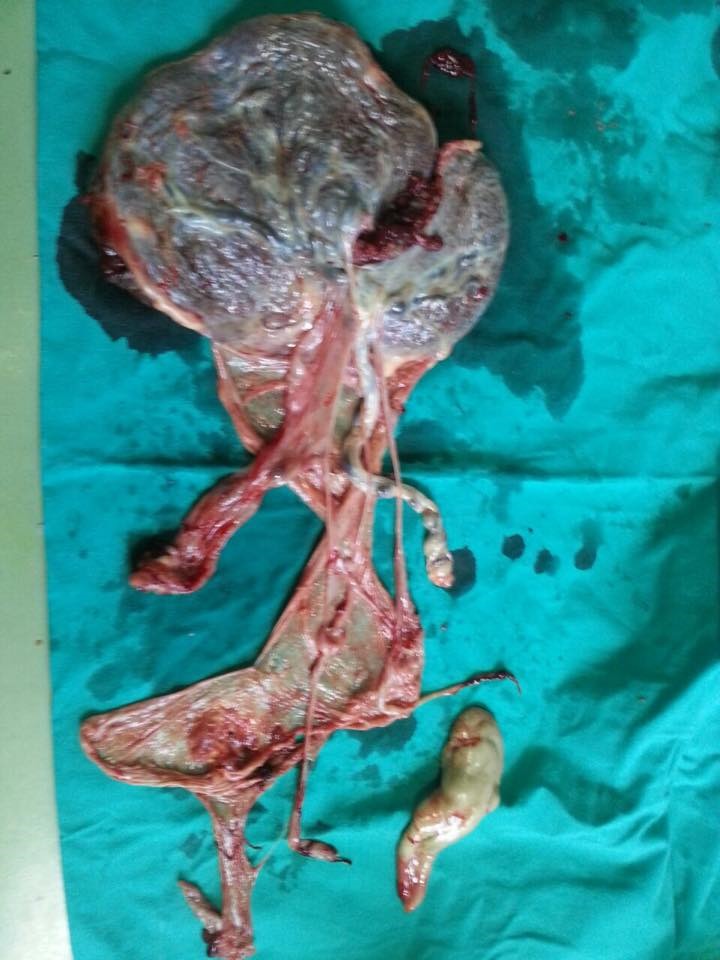
Acardiac twin and placenta

## Discussions

The incidence of TRAP sequence is of 1% in cases of monozygotic twin pregnancies (1 to 35000 births) [**1**]. The physiopathology of this rare complication is uncertain. From the point of view the fetal blood flow, it could be observed that the low oxygen blood from the normal fetus (pumping twin) passed through the placenta arterio-arterial anastomoses to the umbilical artery, and, eventually, in the systemic blood flow of the co-twin recipient (acardiac twin), thus creating a reversed arterial perfusion (TRAP) [**2**]. The pumping twin tried to maintain the pattern of fetal blood flow, the heart of the donor fetus had to support not only its own blood flow but also the acardiac twin’s one. The acardiac fetus did not have a functional heart, so, the blood pressure of the blood received from the donor was higher than the one of the receiver fetus. The presence of arterio-arterial anastomoses allowed the blood to be pumped from the pumping twin to the acardiac twin without passing through a capillary bed. Moreover, veno-venous and arterio-venous anastomoses could appear. The presence of placenta vascular anastomoses is common in monochorionic twins and alone it is not enough to develop TRAP [**2**]. The pattern of abnormal fetal blood flow allowed the placement of a perfusion with unoxygenated blood from the pumping twin at the level of the inferior half of the acardiac twin through its iliac arteries, but also a weak perfusion at the level of the superior part of the body and the head. The acardiac twin had a developed inferior extremity; the pelvis and abdomen were an amorphous mass in which the rest of the fetal organs could not be detected. Another characteristic was represented by the absence of the head or its presence but with malformations (anencephaly or holoprosencephaly), etc. The blood received by the acardiac twin was unoxygenated, poor in nutrients, which came from the apparently healthy fetus. The pumping twin was permanently under a high stress, especially cardiac stress [**3**]. In the case of the TRAP acardiac twin, we had to deal with a parasite fetus whose following parts of the body and organs were not developed: head, arms, and heart. 

**The classification of acardiac twinning was the following**:

**• Hemiacardius** – if the heart is incompletely formed

**• Holoacardius** – if the heart is absent

**Another type of classification was the following**:

**• Acardius anceps **– when the head is poorly formed

**• Acardius acephalus **– if the head is absent

**• Acardius acormus **– presence of head only

**• Acardius amorphous** – unrecognizable anatomy

Regarding the prenatal diagnosis, the main role is represented by the conventional 3/4D Doppler sonography. The acardiac twin – TRAP should be suspected in monochorionic twin pregnancies in which one of the fetuses is normally developed and in the other fetus, no structure or cardiac activity can be detected. The performance of karyotype for the pumping twin is mandatory. 

The indicators of an unfavorable prognosis are the following [**1**,**3**]: 

- The report between the weight of the acardiac twin/ pumping twin higher than 0,70 of the weight of the acardiac twin (in cases in which the value exceeded 0,70, the risk of premature, hydramnios birth was significantly increased) 

- Polyhydramnios (the biggest pocket > 8cm)

- Cardiac failure in the pumping twin can be evidenced by Doppler sonography (diastolic wave inversed on the umbilical artery, flow inversed on the ductus venosum) 

- A growth in dimension of the acardiac twin (abdominal circumference)

- Fetal hydrops in the pumping twin

The evolution without any intervention presents associated risks: intrauterine death (25%), polyhydramnios (50%), premature birth (80%). The survival rate without any intervention is of approximately 55% [**3**]. The signs of cardiac failure in the pumping twin are the following: hydramnios, cardiomegaly, ascitis, and tricuspid regurgitation [**4**]. 

The management implies a periodic ultrasound examination of the pumping twin in order to early detect the signs of fetal hydrops or the alternation of the flows. The administration of corticotherapy between 24 weeks and 34 weeks is important, given the risk of premature birth. Over the years, many efforts have been made to treat TRAP. The interventions that can be performed are the following: laser ablation, bipolar cord coagulation, radiofrequency ablation (RFA). If it is decided that intrauterine surgery is needed, there are two types of approach, such as umbilical cord occlusion – by bipolar procedure (between 17 and 25 weeks of pregnancy)/ ligature (after 26 weeks of pregnancy) and selective fetal reduction [**1**,**5**,**6**]. Both methods can be approached while performing a fetoscopy under ultrasound guidance, but there is no clear data regarding the optimum time for the intervention. Both methods present similar success rates. The maneuver is chosen according to the experience of the surgeon, the gestational age and last, but not least, the choice itself. Fetuses between 18 and 27 weeks of pregnancy, which are prone to a bad prognosis, are the main candidates for the surgery. In a study undergone by Lee et al., in 2007, a survival rate of over 90% of the healthy fetuses after the radiofrequency fetal reduction of acardiac twins was reported. The aim of the treatment was to find a way to safely stop the blood flow to the acardiac twin, so that to assure the safety of the pumping twin and to remove the danger of cardiac failure or death as much as possible. As far as the birth behavior is concerned, an essential issue was to avoid vaginal birth. 

## Conclusions

TRAP should be suspected in cases of monozygous twin pregnancies when no structure/ cardiac activity is noticed in one of the twins while the other one is normally developed (Doppler sonography is compulsory). Cardiac complications, which can lead to cardiac failure, appear early during pregnancy. 
